# Disease the Monitor
*"The Lessons of Acute Disease." By H. Cameron Gillies, M.B. 1892. Price 1£. nett. (London: David Nutt, 270 and 271, Strand.)


**Published:** 1892-09-03

**Authors:** 


### Disease the Monitor.*

"How do you feel after that bad illness of yours?" "Better than I have done for years; it cleared it out of me." Some such conversation takes place between acquaintances every day. It might be difficult for either to say what the "it" was that the illness in question cleared out; but the general meaning is clear—that the ex-invalid is better after his illness than he was before. Formerly he felt fatigue, lassitude, headache, dizziness; now he is free from all these symptoms, he is fresh, active, buoyant, and he gives all the credit to the illness—the fever or pleurisy, the rheumatism or ague, which confined him to his bed for weeks. To say how organic disease can make anyone more healthy would certainly pass the wit of man, but if one must explain phenomena most people will prefer to take the next preceding circumstance as the cause rather than go back to a further incident which carries with it an undeniable reproach. For, in truth, nature has been saying to our ex-invalid friend, "You are using yourself up too fast; I can't keep pace with you. I cannot supply tissue, blood, brain as fast as you use them; do stop and let me make up on you." The man has answered—in deed if not in word—"I can't stop; I want to marry, and have not a sufficient income; I want to set up a carriage; I want to provide for my children; I love the excitement of work and money-making, and I won't stop." Then it comes to a duel between man and nature, and nature wins. Man has only his will—a powerful weapon enough, but not omnipotent; nature has the support of every muscle, nerve, and cell in the body. Nature, Dame Nature, conquers, as women do, by sheer inertia, by refusing to comply with the impossible requirements of masculine will, by sitting down and weeping. Nature's tears are—disease.

In the old legend, Death gave the farmer three warnings of his coming—deafness, blindness, and lameness. The farmer had not understood him, but that was his own fault; Death refused to grant him a longer delay because of his stupidity. Disease acts in much the same way as his sterner brother, with this difference, that if we take heed of its warnings, we may escape a more prolonged visitation. When the monitions of fatigue, headache, dyspepsia, sleeplessness, or whatever form of warning Nature may send are heeded—when sufficient rest, suitable food, pure air, and an absence of excitement are granted to the starved and overworked organism that craves for them, disease in its acuter forms passes by the door; when these warnings are neglected, it comes in and compels the rest the patient would fain refuse to take. You may "work off" a headache for the time, but you cannot work off a fever; you must lie down, and let it take its course. When, after six weeks or so you return to work, feeling as if a burden had been lifted from you, you give the fever the credit. "It cleared 'it' out of me," you declare—whatever the mystic "it" may be. Say rather the fever forced you to rest, and gave nature time to put "it"—arrears of energy—into you. Disease is not in itself a good, but it may be an opportunity for good to be done.

This is the lesson of disease, as Dr. Cameron Gillies strenuously points out; happy are they who profit by it! Few of us, unfortunately, are wise enough to take the milder warnings. We don't stop work for a day because we feel "out of sorts," we will not lay down the pen for a headache, nor give up the rush to the City for a slight feeling of giddiness; so in the end we have six weeks of acute rheumatism, or brain fever, and six months of forced inaction, or apoplexy, brief in its course, indeed, because the end is death. But these were the result of a special accident, it is explained, a chill or a wetting, a business worry, or a mental shock. It is true that some such accident was the final cause; but it was not accident that brought the organism to such a state that an accident would have such serious consequences. It is not the last straw that breaks the camel's back; that straw weighed no more than any other. It is the accumulated weight of all the straws that does the mischief; don't quarrel with the straw, but cease the practice of overloading.

But how are we to know the point at which overloading begins? If we were all as perfect in our fashion as the famous "one hoss shay" it might be difficult to tell; but then in all probability we should go on happily through a long and useful life, and reach our dissolution with the same painless and perfect completeness as that famous vehicle. As it is we really die in pieces, one part giving way before the others, and though "patched-up," as we term it, never becoming as strong as the rest. We have all a weak point. But in place of moaning over this weak point, or trying by all manner of empirical means to deaden its sensations, we might more profitably use it as a barometer, to tell us when our strength is being over strained. A headache, a sore throat, an attack of neuralgia, may be nothing great in themselves, but they indicate a condition which may lead to serious results. Most people try merely to get rid of the pain. Now wet towels, camphorated oil and mustard, even the subtle hypodermic needle injecting a narcotic dose, have their uses; but it is putting them to the worst of ends to employ them merely to deaden the most valuable monitor of our state of health. Skin-deep remedies do little for a constitutional disease. Some people go on employing them, and even make a virtue of their "not giving in." An early gravestone usually tells their tale. It is not, as a rule, the doctors who are to blame. Every text-book of medicine insists on the importance of not treating symptoms, but dealing with the general health; and almost every patient insists on going on in a way which ruins his health, while he demands that all the symptoms of disease shall be either cured or deadened.

This state of affairs will go on—to the profit and delight of vendors of quack medicines and workers of miraculous cures—so long as we go on in the unphilosophical notion

* "The Lessons of Acute Disease." By H. Cameron Gillies, M.B. 1892. Price 1s. nett. (London: David Nutt, 270 and 271, Strand.)

that pain is an evil in itself, and not an indicator of evil. We act towards it like those tyrannic kings of old, who killed the messenger of evil tidings. To strangle pain with narcotics or stimulants is to treat it with an injustice for which ourselves must pay a monitor and guide divinely sent. As surely as hunger and thirst, which are pains also if too long prolonged, imply the need for food and drink, so surely does every pain imply a want in the system which must be satisfied. The Arab may quiet his gnawing stomach for a time by tightening his belt, but he has not thereby replaced the loss he has sustained. He may go on a little longer through this device, but the exhaustion will be the more complete when he breaks down. Nothing is to be gained by ignoring pain; much is lost by stifling it and the tale it tells. The wisest course is to regard it as the localised voice of the whole organism; and to give that organism—not merely the complaining part of it—the treatment (rest, food, air, exercise, change of occupation, or whatever it may be) which we know, though we often do not admit it, that it needs. Thus regarded, disease is the monitor that points the way back to health.

